# Caveolin-1 is down-regulated in alveolar rhabdomyosarcomas and negatively regulates tumor growth

**DOI:** 10.18632/oncotarget.2403

**Published:** 2014-08-27

**Authors:** Juan Huertas-Martínez, Santiago Rello-Varona, David Herrero-Martín, Ignasi Barrau, Silvia García-Monclús, Miguel Sáinz-Jaspeado, Laura Lagares-Tena, Yaiza Núñez-Álvarez, Silvia Mateo-Lozano, Jaume Mora, Josep Roma, Nuria Toran, Sebastian Moran, Roser López-Alemany, Soledad Gallego, Manel Esteller, Miguel A. Peinado, Muro Xavier García del, Oscar M. Tirado

**Affiliations:** ^1^ Sarcoma research group, Molecular Oncology Lab, Bellvitge Biomedical Research Institute (IDIBELL), L’Hospitalet de Llobregat, Barcelona, Spain; ^2^ Developmental Tumor Biology Laboratory, Hospital Sant Joan de Deu, Barcelona, Spain; ^3^ Biomedical Research Unit, Hospital Universitari Vall d’Hebron, Barcelona, Spain; ^4^ Cancer Epigenetics and Biology Programme (PEBC), Bellvitge Biomedical Research Institute (IDIBELL), L’ Hospitalet de Llobregat, Barcelona, Spain; ^5^ Institut de Medicina Predictiva i Personalitzada del Càncer, Badalona, Barcelona, Spain

**Keywords:** alveolar rhabdomyosarcoma, Caveolin-1, muscular differentiation, 5-AZA-2′-deoxycytidine, epigenetics, cell death

## Abstract

Rhabdomyosarcoma is the most common soft tissue sarcoma of childhood and adolescence. Despite advances in therapy, patients with histological variant of rhabdomyosarcoma known as alveolar rhabdomyosarcoma (ARMS) have a 5-year survival of less than 30%. Caveolin-1 (CAV1), encoding the structural component of cellular caveolae, is a suggested tumor suppressor gene involved in cell signaling. In the present study we report that compared to other forms of rhabdomyosarcoma (RMS) CAV1 expression is either undetectable or very low in ARMS cell lines and tumor samples. DNA methylation analysis of the promoter region and azacytidine-induced re-expression suggest the involvement of epigenetic mechanisms in the silencing of CAV1. Reintroduction of CAV1 in three of these cell lines impairs their clonogenic capacity and promotes features of muscular differentiation. *In vitro*, CAV1-expressing cells show high expression of Caveolin-3 (CAV3), a muscular differentiation marker. Blockade of MAPK signaling is also observed. *In vivo*, CAV1-expressing xenografts show growth delay, features of muscular differentiation and increased cell death. In summary, our results suggest that CAV1 could function as a potent tumor suppressor in ARMS tumors. Inhibition of CAV1 function therefore, could contribute to aberrant cell proliferation, leading to ARMS development.

## INTRODUCTION

Rhabdomyosarcoma (RMS) is a rare soft tissue sarcoma, more frequent in children, accounting for 3-4% of childhood cancers. It is believed to be caused by the disruption of regulatory mechanisms that lead to the myogenic phenotype in primitive mesenchymal stem cells [1]. RMS comprises two histological subtypes, alveolar (ARMS) and embryonal (ERMS), each of them with different prognosis and various genetic and molecular alterations. ERMS typically occurs in the head, neck and genitourinary sites and is associated with loss of heterozygosity on the short arm of chromosome 11 (the 11p15.5 region) which codify for various tumor suppressor genes [2-3] imprinted in physiological but not in pathologic conditions. ARMS, on the other hand, commonly arises in the trunk and extremities and is linked with acquired specific chromosomal translocations t(2;13)(q35;q14) or t(1;13)(p36;q14) in 70 to 85% of cases. These translocations give rise to the fusion of the PAX3 or PAX7 transcription factor to the forkhead (FOXO1) transcription factor. All characterized PAX-FOXO1 chromosomal translocations generate structurally equivalent, in-frame PAX-FOXO1 chimeric transcription factors, where the PAX paired box and homeodomain DNA-binding domains are fused to the transcriptional activation domain of FOXO1 [4]. PAX3/7-FOXO1 aberrant fusion proteins behave as oncoproteins deregulating PAX3 and PAX7 transcription factor networks that play a role in skeletal muscle development, thus altering aspects of the muscle development, growth and/or maintenance. In the end PAX/FOXO1 proteins drive neoplastic transformation of skeletal muscle lineage cells towards malignant, developmentally arrested primitive myoblasts [5].

Caveolin-1 (CAV1) is the principal structural protein responsible for the formation of caveolae in the cell membrane. The capacity of CAV1 to associate with a wide variety of proteins is crucial in a number of processes, ranging from vesicular transport and cholesterol homeostasis to nitric oxide production and cell migration, among others [6-9]. CAV1 has been thoroughly characterized in many cancers due to its ability to regulate cell cycle progression and intracellular signal transduction, and it has been shown to act both as a tumor suppressor or tumor promoter depending on the cellular background [10-12].

In a recent study, it was shown that CAV1 was predominantly expressed in the ERMS histotype and placed CAV1 as a valuable marker of diagnosis for RMS characterized by low degree of differentiation [13]. We further confirmed the absence of CAV1 in ARMS cell lines [12]. However, the mechanism responsible for CAV1 absence and its putative role in ARMS as a tumor suppressor has not been investigated yet. We show here that CAV1 silencing in ARMS cells is a consequence of promoter methylation. Additionally, we show that re-expression of CAV1 in three of these cell lines is cytostatic and promotes features of muscular differentiation. *In vivo*, CAV1-expressing tumor cells show growth delay and higher muscular differentiation in comparison to untransfected and vector transfected cells. Our results demonstrate that CAV1 could function as a potent tumor suppressor in ARMS tumors. Inhibition of CAV1 function could contribute to aberrant cell proliferation, leading to ARMS development.

## RESULTS

### CAV1 is down-regulated in ARMS cell lines and tumor samples

**Figure 1:**
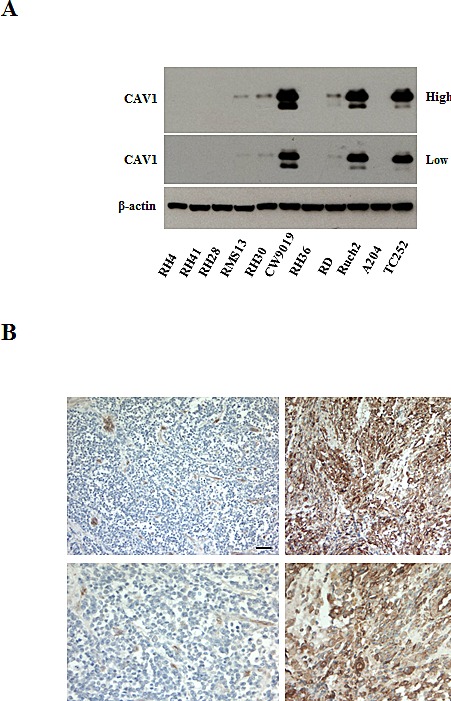
CAV1 expression in rhabdomyosarcomas (A) Western blot showing CAV1 expression (at high and low exposure) in different ARMS (RH4, RH41, RH28, RMS13 RH30 -PAX3/FOXO1- and CW9019 -PAX7/FOXO1-), ERMS (RH36, RD), Botryoid (RUCH2) and Rhabdoid (A204) cell lines, using a Ewing sarcoma cell line (TC252) as a positive control, (B) CAV1 staining from a pleural ARMS sample (right panel), representative of the absence of CAV1 expression in these patients, and from a pelvic ERMS in stage 4 (left panel), representative of high expression of CAV1. Scale bars 100 μm and 50 μm for the higher magnification.

We and others have previously shown down-regulation of CAV1 in ARMS cell lines [12-13]. To further determine the expression levels of CAV1 in RMS cell lines and patients, we analyzed its expression by western blot in a panel of human cell lines and by immunohistochemistry (IHC) in a tissue microarray (TMA) of 70 patients (Figure 1 and Supplementary Table S1). Most of the ARMS cell lines tested had very low or undetectable levels of CAV1 (Figure 1A). In the TMA (Figure 1B), CAV1 expression was detected mostly in ERMS patients (28/39) and was localized in the cytosol and plasma membrane. Barely any expression was found in ARMS patients (2/12). Expression in other forms of RMS was variable (9/19). As shown in supplementary Table S1, not enough clinical information was available to establish any significant statistical correlation.

### CAV1 is silenced in ARMS cells by epigenetic mechanisms

**Figure 2:**
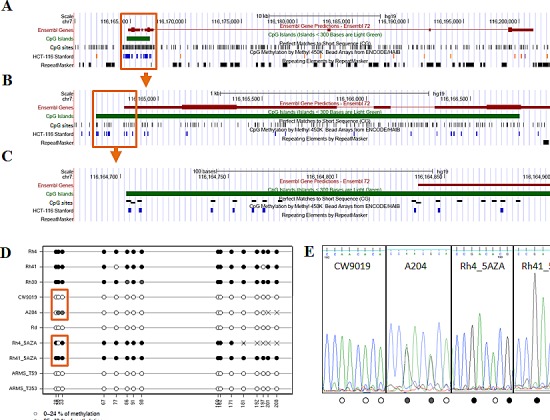
Analysis of DNA methylation in the gene promoter (A) Representation of the *CAV1* gene in the UCSC Genome Browser. The promoter-associated CpG island is boxed. The tracks indicate the position of CpG sites and Infinium 450K methylation arrays (HCT-116 Stanford), (B) Enlarged display of the CpG island. The region analyzed for DNA methylation is boxed, (C) Region analyzed for DNA methylation by bisulfite sequencing, (D) DNA methylation levels of the CpGs analyzed by bisulfite sequencing (E) Detail of the electropherogram showing three CpGs differentially methylated enclosed in a box in panel D. Methylation level of each CpG is indicated at the bottom of the sequence (reverse strand) using the same code of panel D, RT-PCR (F) and Western Blot (G) 72h after 5-aza-dC treatment in RH4 cells.

CAV1 has been reported to be up-regulated by the DNA methyltransferase inhibitor 5-AZA-2′-deoxycytidine (5-aza-dC) in several types of cancer [14]. Moreover, hypermethylation of the *CAV1* promoter in human cancer has also been shown [15-17]. Analysis of DNA methylation in the *CAV1* gene promoter (Figure 2A-C) of RMS cell lines and tumor samples showed hypermethylation in the promoter-associated CpG island in 5 out of 6 ARMS cell lines but in any of the other cell lines used in the experiment nor in the two ARMS tumor samples (Figure 2D-E and Supplementary Figure S1). Treatment of RH4 cells with increasing concentrations of 5-aza-dC induced re-expression of *CAV1* mRNA and protein (Figure 2F and G). Similar results were obtained in other three ARMS cell lines (RH41, RH28 and RMS13) (Supplementary Figure S2).

### Over-expression of CAV1 suppresses tumorigenicity of ARMS cells

**Figure 3:**
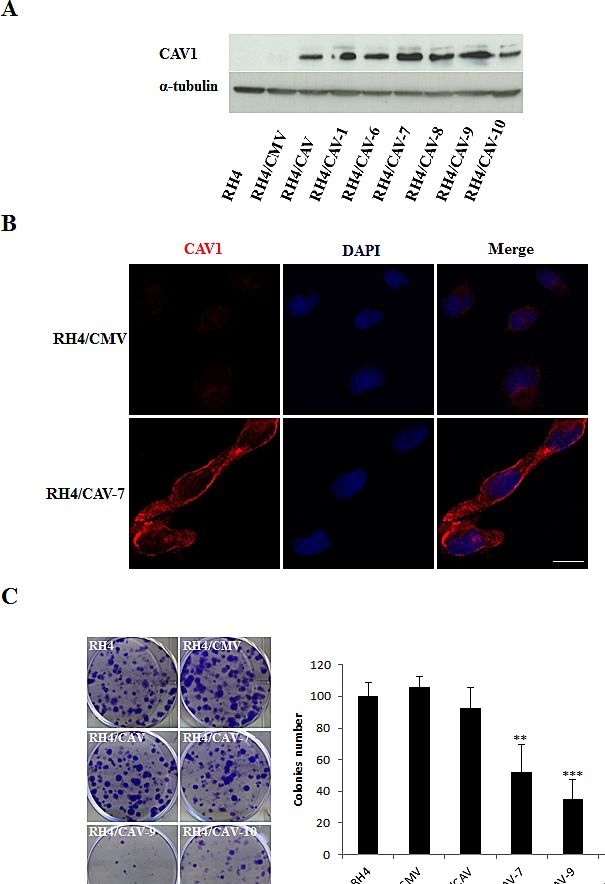
Effects of CAV1 transfection in the RH4 cell line (A) Western Blot showing expression of CAV1 on isolated clones, (B) Inmunofluorescence showing expression and localization of CAV1 in the plasma membrane of RH4 transfected cells, Scale bars 10μm, (C) Clonogenic assay using the RH4 model showing a decrease in the clonogenic capacity in the CAV1 transfected cells (CMV stands for empty vector transfected cells and CAV refers to CAV1 transfected cells, the number indicates the clone). Statistical significance was assessed by the Student’s *t* test: **p ≤ 0.001 and ***p≤0.0001.

**Figure 4:**
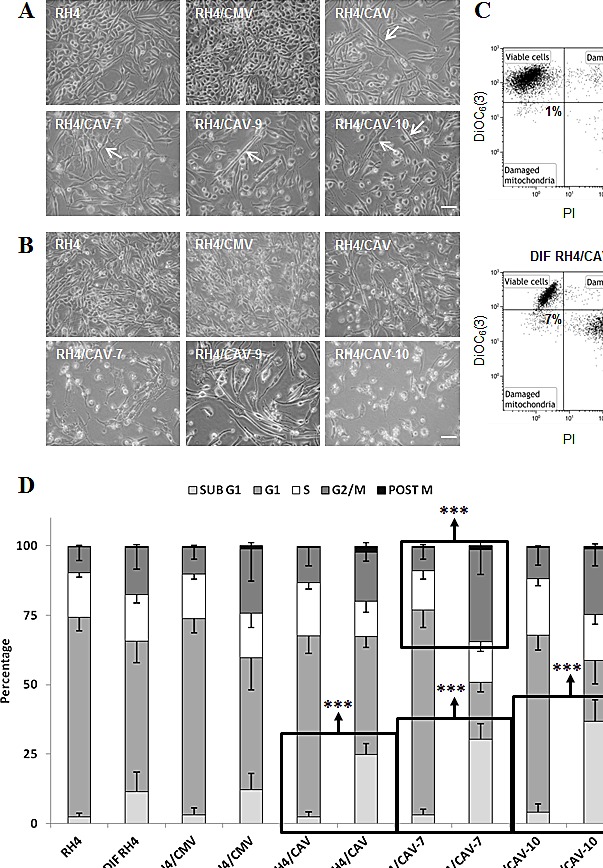
RH4 rhabdomyosarcoma cells expressing CAV1 show an increased capacity for initiate differentiation process, but they die before fully completing it (A) CAV1 expressing RH4 cells cultured in differentiation media (RPMI medium without serum) for 72 h change their morphology by the acquisition of an elongate form (arrows), (B) Wild-type RH4 rhabdomyosarcoma cells grow normally in differentiation conditions up to 120 h, but transfected cells expressing CAV1 die, (C) Cytofluorometric plots showing the acquisition of the vital dye DiOC_6_(3) which accumulates in mitochondria maintaining their characteristic membrane potential (Δψ_m_) and the cell death marker Propidium Iodide (PI) who only enters in cells whose plasma membrane barrier is broken. RH4/CAV-10 population shows a clear shift towards losing both the Δψ_m_ and the membrane integrity, hallmarks of apoptosis, (D) Cell cycle analysis by means of cytofluorometric measurement of DNA-binding dye PI content in fixed cells. Cells cultured in differentiation conditions show a trend to be arrested in G_2_/M phase of the cell cycle. The G_2_/M blockade becomes significant in RH4/CAV-7 line. CAV1 expressing cells show a marked increase of the number of cells with a DNA content inferior of the G_1_ phase (apoptotic). Statistical significance was assessed by the Student’s *t* test: ***p ≤ 0.0001. Scale bars 50 μm.

In order to explore the role of CAV1 in the progression of ARMS we stably transfected RH4, RH41 and RH28 cells with the expression vector pCMV6-CAV1. Over-expression of CAV1 was confirmed in several selected clones by western blot (Figure 3A, Supplementary Figure S3A and Supplementary Figure S4). Changes in CAV1 protein expression were also confirmed by immunofluorescence, where over-expressing cells demonstrated increased cytoplasm and membrane localization of CAV1 following transfection (Figure 3B). Moreover, as a result of CAV1 reintroduction, clonogenic growth was significantly affected (Figure 3C and Figure S3B). It is well known that the biological behavior of a tumor is related to the degree of differentiation of its cells, and a lower degree of differentiation generally correlates with greater tumor growth. Accordingly, as a consequence of CAV1 transfection we observed elongated cell morphology and appearance of cross-striations in some cells, consistent with a more differentiated myogenic phenotype (Figure 4A). This effect was further highlighted in differentiation conditions on RH4 and RH28 models (Figure 4B and Supplemental Figure S5A). Additionally, under differentiation conditions most of CAV1 transfected cells failed to maintain the polarization of the outer mitochondrial membrane and the barrier of the plasma membrane (as visualized by cytofluorometric analysis with the probes DiOC_6_(3) and Propididum Iodide (PI) (Figure 4C and Supplemental Figure S5B). When cultivated in differentiation conditions (Figure 4D, Supplementary Figure S5C and Supplementary Figure S6), CAV1-transfected cells significantly increased the amount of apoptotic cells. More interestingly, differentiation conditions led to an increase of G_2_/M cells (assumed as G_2_ cells as microscopical observation showed not dividing cells) especially significant in clone 7. Combination of the DiOC_6_(3)-PI viability assay with the cell permeable DNA dye Hoechst-33342 pointed that at least part of the dying cells start apoptosis from G_2_ phase (data not shown).

**Figure 5:**
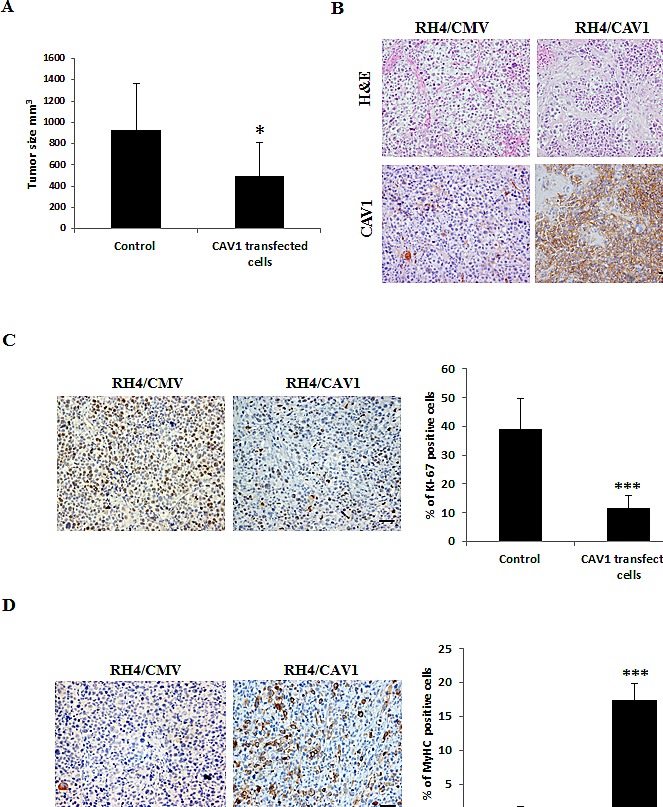
CAV1 delays ARMS *in vivo* tumor growth (A) Graphic comparing tumor size (mm^3^) in the RH4/CAV1 model (Control states for RH4 and RH4/CMV, CAV1 transfected cells states for RH4/CAV-7 and RH4/CAV). CAV1 expressing cells grow significantly less than control cells, (B) Histopathology was examined by hematoxilin/eosin (H&E) and CAV1 expression by IHC staining, (C) proliferative capacity of the tumors was evaluated by Ki-67 staining. Positive cells were counted and percentage represented in the graphic, (D) differentiation state of the tumor was evaluated by MyHC staining. Positive cells were counted and percentage was represented in the graphic. Statistical significance was assessed by the Student’s *t* test: *** p<0.0001. Scale bars 50 μm.

Most importantly, *in vivo* experiments on the RH4 model showed that, 40 days after s.c. injection into nude mice, CAV1–derived xenografts were significantly smaller (*p* ≤ 0.05) than those induced by control cells (Figure 5A). Immunohistochemical analyses of paraffin-embedded tumors showed no detectable CAV1 expression in control xenografts compared with the highly positive staining of CAV1–derived tumors (Figure 5B). Interestingly, CAV1-derived xenografts showed significant less Ki-67, a known marker of proliferation. On the other hand CAV1-derived xenografts showed more Myosin Heavy Chain (MyHC) staining (Figure 5C-D) suggesting tumors are less proliferative and more prone to differentiate. Altogether, these data show that CAV1 is a key negative effector of tumorogenesis, necessary for the development of the transformed phenotype in ARMS sarcomagenesis.

**Figure 6:**
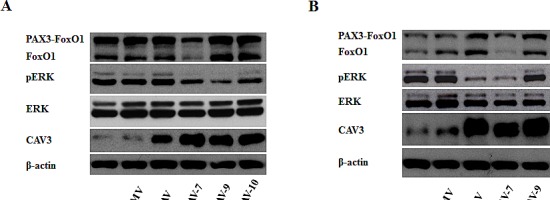
CAV1 affects ERK phosphorylation and promotes changes in CAV3 (A) Western blot showing PAX3-FOXO1, FOXO1, CAV1, ERK, phospho-ERK and CAV3 levels in cells grown in proliferating conditions or (B) grown under differentiation conditions.

Because PAX/FOXO1 proteins block terminal differentiation in ARMS [5], we analyzed possible changes in these proteins as a consequence of CAV1 transfection. No significant changes were observed under proliferation (Figure 6A) or under differentiation conditions (Figure 6B). However, in clones 7 and 10, where higher phenotypic alterations were observed, under differentiation conditions FOXO1 levels were decreased. ERK dephosphorylation is key at the onset of myogenesis [14]. Furthermore, CAV1 is known to block MAPK signaling as part of its tumor suppressive activities [15]. Therefore, in order to go deeply into the mechanism by which CAV1 delays tumor growth in ARMS we sought to analyze the effects of CAV1 expression on MAPK signaling. Results showed that indeed, CAV1 expression caused ERK dephosphorylation under proliferating conditions (Figure 6A). This effect was further accentuated in the majority of the clones, except for clone 9, when cells were grown under differentiation conditions (Figure 6B). Moreover, a known marker of differentiation, CAV3, was increased both under proliferation and differentiation conditions (Figure 6A and 6B).

In order to demonstrate the CAV1 dependent pro-differentiation effects on ARMS cells we tested the modulation of myogenin in the early and MyHC in the late phase of differentiation. As shown in Supplementary Figure S7, in comparison to controls CAV1 transfected cells showed an upregulation of myogenin up to 72 hours followed by an important decrease at 120 hours. MyHC appeared mostly at 120 hours (Supplementary Figure S7).

Overall our results strongly suggest that CAV1 expression delays ARMS cell growth, at least in part, by blocking ERK signaling, turning malignant cells toward a more permissive state for differentiation and cell death.

## DISCUSSION

Testing for the expression of CAV1 in several sarcoma cell lines we found that its expression in most alveolar rhabdomyosarcoma cells was very low or undetectable [12]. Because proliferating myoblasts express CAV1 [16], we postulated that very low or no expression on proliferating tumor cells suggest that CAV1 would have suppressive activities on ARMS. In fact, a tumor suppressor role for CAV1 has been clearly demonstrated in several types of cancer [17-19] including sarcomas [20]. To test our hypothesis we validated first the expression levels of CAV1 on a broader number of cell lines and in tumor samples. Results confirmed that most ARMS cell lines, except for CW9019 (PAX7-FOXO1), and patients presented barely any expression of CAV1. This result might suggest that downregulation of CAV1 correlates specifically with PAX3-FOX1 expression. Unfortunately, we did not possess exact information about the type of translocation in our ARMS patients cohort. Analysis of this issue in further patient cohorts will help to elucidate whether this relationship exist.

CAV1 can be negatively regulated by several means. For example, DNA methylation, that is an epigenetic mechanism of transcriptional regulation. Deregulation of DNA methylation in cancer results in inappropriate silencing of tumor suppressor genes by hypermethylation of the promoter region and oncogene overexpression by loss of methylation [21-23]. Thus, hypermethylation of CAV1 promoter has been shown to maintain it at low expression levels in some types of cancer such as breast and colon [24-25]. Therefore, we tested this possibility in several cell lines and tumor samples. Bisulfite sequencing showed heavy hypermethylation of the CAV1 promoter in ARMS cell lines. However, although no expression of CAV1 was detected in tumor samples no methylation was observed in its promoter, suggesting that promoter hypermethylation is a secondary event to gene silencing which would be induced by other mechanisms [26]. In this regard, one possibility is that expression of CAV1 might be regulated by microRNAs (miRNAs). In fact several miRNAs such as miR-124, miR-203, miR-199a and miR-802 had been found to directly suppress CAV1 in other tumors [27-31]. Among them miR-199a is the only one found highly expressed in ARMS [32] suggesting that this miRNA could be a good candidate as responsible for CAV1 downregulation in the tumor samples studied. Whether miR-199a targets CAV1 in ARMS deserves therefore further work. Another possibility is proteasomal degradation. Actually, CAV1 had also been shown to be degraded by the proteasome [33]. Hence, activity of stromal factors acting on proteasome pathways in tumor cells can occur in ARMS patients and be responsible of CAV1 downregulation.

Our gain of function experiments clearly demonstrated that CAV1 had suppressive activities in ARMS cells both *in vitro* and *in vivo*, effects that were highlighted when cells were grown under differentiation conditions. Mitochondrial membrane potential (Δψ_m_) dissipation is a well known process in apoptosis signaling, whereas the influx of Propidium Iodide inside the cells is a sign of loss of cationic pumps shared by late apoptotic cells and necrotic ones. Different end-point analyses (data not shown) suggests that the dissipation of the mitochondrial membrane potential precedes the loss of the plasma membrane barrier, indicating that CAV1 transfected cells triggered apoptosis in differentiation conditions.

One of the key features of ARMS is its incapacity to proceed through terminal differentiation [4]. Furthermore, the degree of myogenic differentiation in these tumors has been shown to inversely correlate with proliferation, migration and invasion [34]. Our results strongly suggest that CAV1 mediates tumor suppression, at least in part, by turning tumor cells more prone to differentiate. Accordingly, CAV3, a marker of differentiation [35] was highly upregulated in CAV1 transfected cells. Likewise, dephosporylation of ERK1/2, necessary for cell fusion [36] prior to myotube formation and higher expression of myosin heavy chain proteins, exclusive of myotubes [37] were also observed. Interestingly, FOXO1 levels were reduced in clones 7 and 10 paralleling phenotypic alterations in cells cultured under differentiation conditions. In agreement with these results, FOXO1 has been shown to delay and negatively regulate skeletal myoblast differentiation [38].

Recently Faggi *et al*. reported that overexpression of CAV1 leads to rhabdomyosarcoma cell proliferation [39], using the RH30 cell line as a model of high expression of CAV1. These reported results argue against the conclusions of our study. Nevertheless, in our panel of cells, RH30 and RMS13 (cells that come from the same patient) showed fairly low levels of CAV1. Furthermore, treatment of RMS13 with 5-aza-dC induced re-expression of *CAV1* mRNA and protein. So, in our opinion this cell line is not representative of highly expressed CAV1 and therefore our panel of ARMS cells reflects better the role of CAV1 as a putative tumor suppressor in ARMS.

In summary, our results suggest that CAV1 could function as a potent tumor suppressor in ARMS tumors. Inhibition of CAV1 function therefore could contribute to aberrant cell proliferation, leading to ARMS development. Finally, we propose that mimicking CAV1 function may be of therapeutic use for the treatment of ARMS.

## MATERIALS AND METHODS

### Cell culture, stable transfection and treatments

RH4 and RH30 (kindly provided by Dr. Peter Houghton, The Research Institute Nationwide Children’s Hospital, Columbus, Ohio), RH28, RMS13, RH36 and Ruch2 (kindly provided by Dr. Beat Schäfer, Department of Oncology and Children’s Research Center, University Children’s Hospital, Zurich, Switzerland), CW9019 (kindly provided by Dr. Frederic Barr, National Cancer Institute, Bethesda, MD, USA), RH41, RD and A204 (bought from DSMZ, Libniz Institute DSMZ-German Collection of Microorganisms and Cell Cultures, Braunschwieg Germany) and TC252 cell lines (kindly provided by Dr. Heinrich Kovar, Children’s Cancer Research Institute (CCRI), Viena, Austria) were cultured in RPMI 1640 (Invitrogen) supplemented with 10% heat-inactivated fetal bovine serum (Invitrogen). All cell lines were incubated at 37°C in a humidified atmosphere of 5% CO2 in air. Exponentially growing cells within two sequential passages were used for all experiments. Cells were transfected using Lipofectamine 2000 (Invitrogen) following the protocols of the manufacturer. Transfected cells were selected with neomycin [0.6 mg/m1 for RH4 0.4 mg/ml for RH41, Invitrogen] for 14 days. pCMV6-CAV1 was bought from OriGene Technologies, Medical Center, Rockville, Maryland. Antibiotic-resistant pools and individual clones were isolated for further analysis and maintained in the presence of neomycin (0.4 mg/ml or 0.6 mg/ml). For differentiation experiments cells were serum deprived for 72h. All cell lines were treated with 5-AZA-2′-deoxycytidine (5-aza-dC) (Sigma) to allow global CpG demethylation. 2×10^5^ cells/well were plated in 6-well dishes and treated after 24h with 1, 2.5 and 5 μM 5-aza-dC for 72h. Drug was added every 24h.

### Bisulfite sequencing

Caveolin 1 CpG island methylation was analyzed by bisulfite sequencing. Briefly, 300 ng of genomic DNA were converted with EZ DNA methylation kit^TM^ (Zymo Research), according to manufacturer’s instructions. Two different fragments spanning a total of 16 CpGs (Figure 2) were amplified by a nested PCR performed in triplicate and pooled before purification using JETQUICK PCR Spin KIT (Genomed), to ensure a representative methylation profile. The PCR products were sequenced using specific primers at GATC Biotech service. The primers used for the PCR amplifications were designed using MethPrimer and Bisearch [40] are: external PCR (GAGGTGGGAAGGGATGGTTTA, AAATTTCCCTAAACTATACTTTAA), internal PCR A (GTTGTTTATATTGGGTATTTTTG, TCTAAACACATCCCCAAAATTC), internal PCR B (ATTTTTGTTGAGATGATGTATTG, TCTAAACACATCCCCAAAATTC). Lollipop representations were generated using the Methylation Plotter web tool [41].

### Clonogenic assay

For clonogenic assays, 500 cells/well were seeded in 6-well plates. When colonies reached saturation (14 days after seeding) cells were fixed with 4% formaldehyde for 30 minutes, washed with Dulbeco’s PBS (DPBS), stained with violet crystal for 20 minutes and washed with water. Plates were scanned and colonies counted. Images reflect representative results of at least three independent experiments.

### Flow cytometry

For the simultaneous quantification of plasma membrane integrity and mitochondrial transmembrane potential (Δψm), living cells were collected and stained with 1 μg/mL propidium iodide (PI, which only incorporates into dead cells, from Molecular Probes) and 40 nM of the Δψ_m_-sensitive dye 3,3′-dihexyloxacarbocyanine iodide (DiOC_6_(3), from Sigma-Aldrich) for 30 min at 37 °C. The cell-permeable DNA-binding dye Hoechst-33342 (Molecular Probes) were also added (10 μg/mL final concentration) for determining the cell-cycle status of live or dying cells.

For cell cycle analysis, cells were fixed in 70% ice-cold ethanol and labeled with 50 μg/ml PI in the presence of 500 μg/ml RNAse (Sigma-Aldrich). Cytofluorometric determinations were performed by means of a Gallios flow cytometer and data were statistically evaluated using Kaluza software (Beckman Coulter). Only the events characterized by normal forward scatter (FSC) and side scatter (SSC) parameters were included in subsequent analyses.

### Western Blot analysis

Cells were lysed with radioimmunoprecipitation assay buffer (RIPA Bufer, Thermo Scientific) containing protease inhibitors (Complete, Mini; Protease Inhibitor Cocktail Tablets, Roche) and phosphatase inhibitors (PhoStop, Phospatase Inhibitor Cocktail Tablets, Roche) and centrifuged at 13000×g, at 4ºC, for 20 minutes. The protein content of the supernatants was determined with BCA assay system (Pierce). Lysate aliquots (50 μg) were resolved by 8%, 10% or 12% (depending on the protein molecular weight) SDS-PAGE and transferred onto nitrocellulose membranes. After blocking with 5% skimmed milk in PBS containing 0.2% Tween 20 at room temperature for 1 hour, membranes were incubated overnight at 4ºC with the appropriate primary antibody (CAV1 #610059 from BD, FoxO1 #2880, ERK1/2 #4695, phospho-ERK1/2 #4376 from Cell Signaling Technology; CAV3 #sc5310 and Myogenin #sc-12732 from Santa Cruz; MyHC MF 20 from Developmental Studies Hybridoma Bank). Blots were then incubated at room temperature for 1 hour with a horseradish peroxidase–conjugated secondary antibody and the peroxidase activity was detected by enhanced chemiluminescence (Pierce) following the instructions of the manufacturer. Immunodetection of β-actin (#ab49900) from Abcam was used as a loading reference.

### Xenografts

*In vivo* tumors were induced with subcutaneous injections of RH4/CAV1 model (3 ×10^6^ cells), resuspended in 100μL of Matrigel Matrix (BD), in the hind legs of 10 athymic nude mice purchased from Charles River (Left flank RH4, right flank RH4/CAV-7 n=5; Left flank RH4/CMV, right flank RH4/CAV n=5). When the tumor reached a mean volume of about 1cm^3^ mice were euthanized and the tumor was removed for further analysis. Tumors were fixed in 4% paraformaldehyde and embedded in paraffin. Tumor volumes were calculated using the formula V= (1/2)a×b^2^, where a is the longest tumor axis, and b is the shortest tumor axis. Data are given as mean ±SD. Statistical analysis was done by unpaired Student’s t test. Animal care procedures were followed according to the Institutional Guidelines for the Care and Use of Laboratory Animals. Ethics approval was provided by the locally appointed ethics committee from the Biomedical Research Institute (IDIBELL), Barcelona, Spain.

### Immunohistochemistry and Immunofluorescence

Immunohistochemical techniques were done as previously described [42]. Expression of CAV1 in xenografts was analyzed using a rabbit polyclonal antibody (CAV1 #610059, BD). Proliferation marker Ki-67 and differentiation marker MyHC were analyzed using rabbit polyclonal antibody (Ki-67 #18-0191Z, Life Technologies) and Mouse monoclonal antibody (eMHC F1652 Developmental Studies Hybridoma Bank). Immunofluorescence of ARMS cells was performed as described [43]. Photographs were taken with a Leica TCS SP5 spectral confocal microscope (argon, 405 diode and DPSS561) using a lambda blue 63×1.35 numerical aperture oil objective. Images were analyzed with Image J software (freely available from the National Institutes of Health (NIH) at the address http://rsb.info.nih.gov/ij/).

### Reverse Transcription-PCR (RT-PCR)

Total RNA (2μg), extracted using the Total RNA Isolation Kit (NucleoSpin RNA II, Macherey-Nagel), was used for cDNA synthesis with SuperScript II Reverse Transcriptase (Invitrogen). Primers 5′- ACAAGCCCAACAACAAGG-3′ (forward) and 5′- ATCGGGATGCCAAAGAGG-3′ (reverse) were used for amplification of *Caveolin-1* (259 bp); and for *β-Actin* (432 bp), 5′- CGGGACCTGACTGACTACCTC-3′ (forward) and 5′- CTTCATTGTGCTGGGTGC-3′ (reverse) from Invitrogen. Amplification of *Caveolin-1* was adjusted at an annealing temperature of 58.4 °C and 59.5 °C for *β-Actin*. For each set of primers, the numbers of cycles was adjusted so that the reaction end points fell within the exponential phase of product amplification, thus providing a semi-quantitative estimate of relative mRNA abundance. RT-PCR determinations were carried out thrice for each relevant transcript.

### Quantitative Real Time PCR

Total RNA was extracted using the Total RNA Isolation Kit (NucleoSpin RNA II, Macherey-Nagel). 2 μg of RNA were used for cDNA synthesis with SuperScript II Reverse Transcriptase (Invitrogen). Quantitative reverse transcription-PCR (qRT-PCR) was performed under universal cycling conditions on an ABI 7300HT instrument (Applied Biosystems) using commercially available CAV1 probe (Hs00971716_m1) and mastermix (all from Life Technologies). Cycle threshold (*C*_T_) values were normalized to Beta Actin (ACTB). Experiments were performed at least three times and in triplicates. Relative expression level of the target gene among the different samples was calculated using the ΔΔC_T_ method [44]. Mean values and standard deviations were calculated based on the results of three biological replicates at least.

### Statistical Analysis

Data were analyzed for statistical significance using Student’s *t* test. Unless otherwise stated, experiments were performed thrice; *P* ≤ 0.05 was regarded as significant.

## SUPPLEMENTARY FIGURES AND TABLE




