# Supplementary material for: Caveolin-1 is down-regulated in alveolar rhabdomyosarcomas and negatively regulates tumor growth
Source: Oncotarget. 2014 Aug 27;5(20):9744–55. doi: 10.18632/oncotarget.2403 (PMC4259434; doi:10.18632/oncotarget.2403)
Supplement: Supplementary file 1 [file oncotarget-05-9744-s001.pdf]

Caveolin-1 is down-regulated in alveolar rhabdomyosarcomas and negatively regulates tumor growth

Supplementary Material

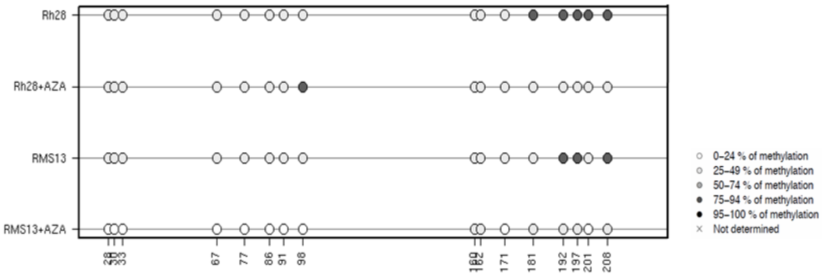

Supplementary Figure S1: DNA methylation levels of the CpGs analyzed by bisulfite sequencing in RH28 and RMS13 cell lines.

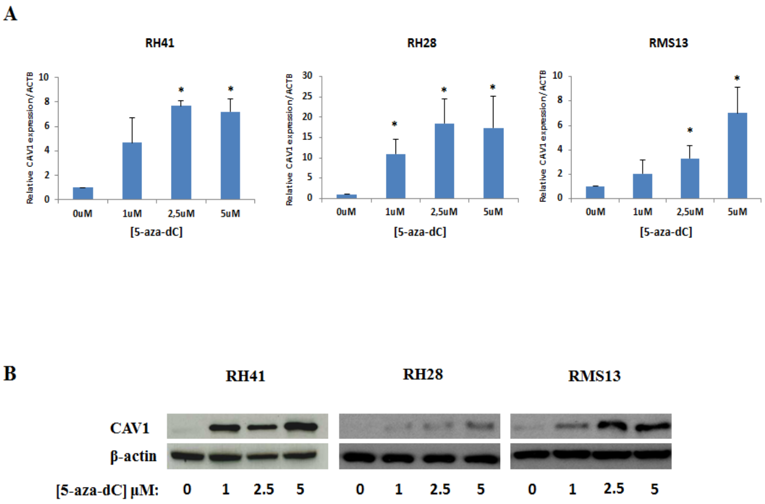

Supplementary Figure S2: qPCR (A) and Western blot (B) 72h after 5-aza-dC treatment in RH41, RH28 and RMS13 cell lines showing expression of CAV1 at mRNA and protein level. * p<0,05 compared to control (Ctrl).

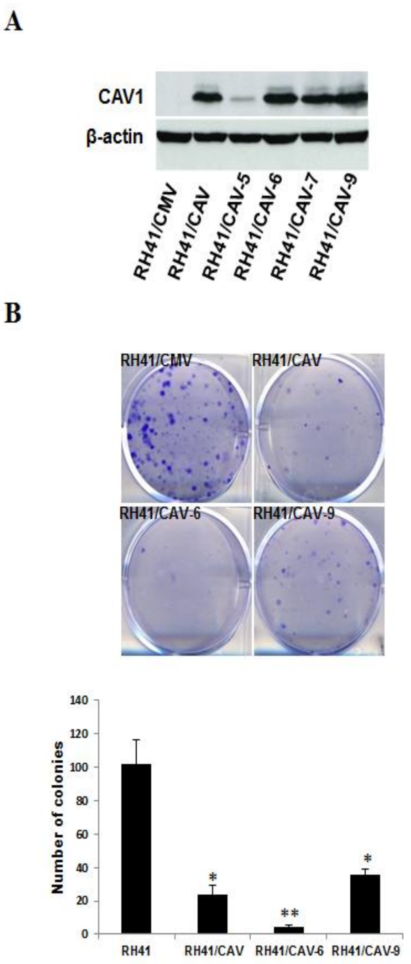

Supplementary Figure S3: Effects of CAV1 transfection in the RH41 cell line. (A) Western blot showing CAV1 expression in the isolated clones, (B) Clonogenic assay using the RH41 model showing a decrease in the clonogenic capacity in the CAV1 transfected cells (CMV stands for empty vector transfected cells and CAV refers to CAV1 transfected cells, the number indicates the clone). Statistical significance was assessed by the Student's *t* test: *p ≤ 0.05 and **p≤0.001).

A

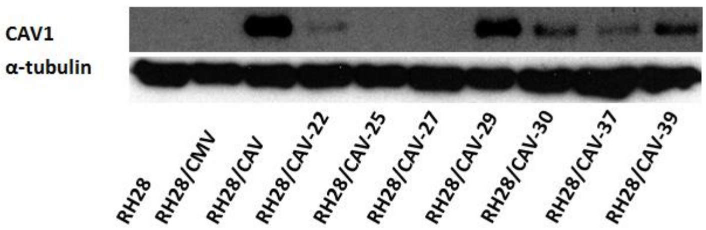

B

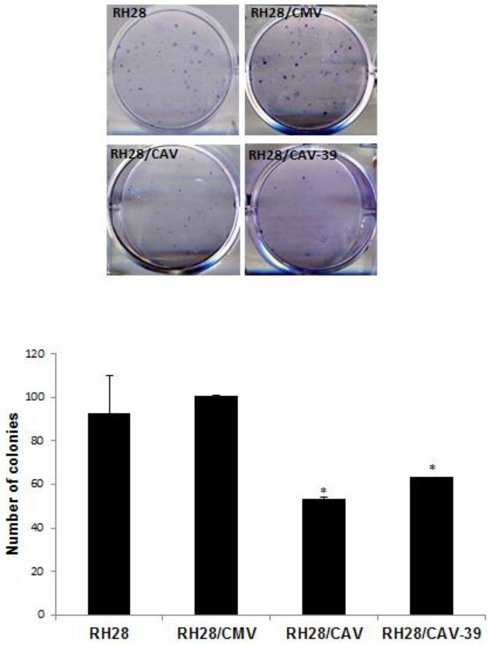

Supplementary Figure S4: Effects of CAV1 transfection in the RH28 cell line. (A) Western Blot showing CAV1 expression in RH28 in the isolated clones, (B) Clonogenic assay using the RH28 model showing a decrease in the clonogenic capacity in the CAV1 transfected cells (CMV stands for empty vector transfected cells and CAV refers to CAV1 transfected cells, the number indicates the clone). Statistical significance was assessed by the Student's *t* test: $*p \leq 0.05$.

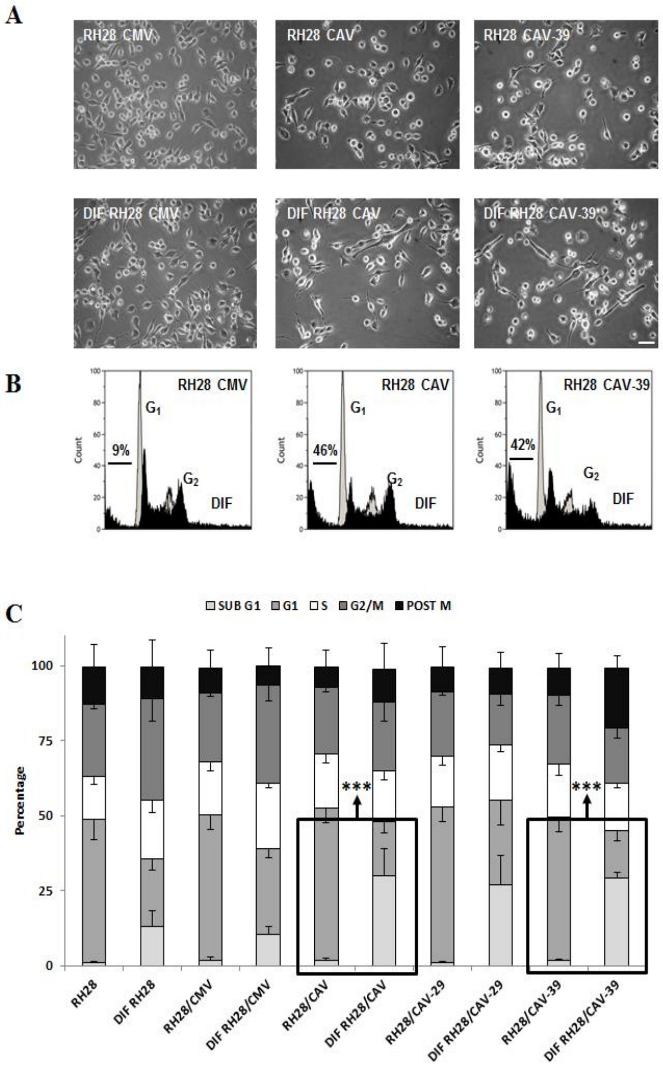

Supplementary Figure S5: RH28 cells expressing CAV1 behaves mostly as RH4 cells transfected with CAV1. (A) RH28 cell expressing CAV1 grow normally in proliferation conditions (upper panel) but when maintained for 120 h in differentiation conditions (RPMI medium without serum) CAV1 expressing cells show an accumulation of detached death cells as well as attached giant differentiated cells. (B) Propidium Iodide staining plots obtained by flow cytometry: CAV1 expressing RH28 cells cultured in differentiation media for 120 h showed an increase in sub-G_1 peak (dark ones) compared to cells growth in normal media (light ones). Differentiated cells showed as well a general shift of the plot to the right, meaning a general increase in DNA-content. (C) Cell cycle analysis by means of cytofluorometric measurement of DNA-binding dye PI content in fixed cells. Cells cultured in differentiation conditions show a trend to be arrested in G_2/M phase of the cell cycle, albeit is minor that the seen in RH4 cells. CAV1 expressing cells show a marked increase of the number of cells with a DNA content inferior of the G_1 phase (apoptotic). Statistical significance was assessed by the Student's *t* test: $***p \leq 0.0001$. Scale bars 50 µm.

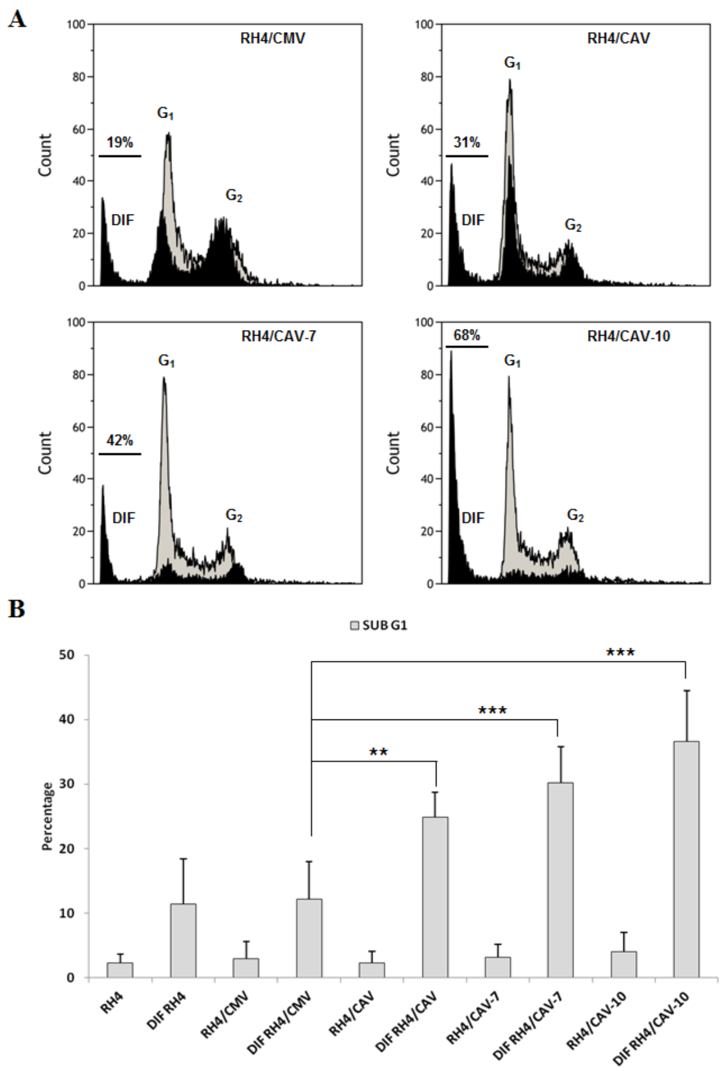

Supplementary Figure S6: RH4 cells expressing CAV1 show an increased capacity for initiate differentiation process, but they die before fully completing it. (A) Propidium Iodide staining plots obtained by flow cytometry: CAV1 expressing RH4 cells cultured in differentiation media for 120 h showed an increase in sub-G_1 peak (dark ones) compared to cells growth in normal media (light ones) (B) Sub-G_1 percentages extracted from Figure 4D. The increase in the amount of apoptotic cells in CAV1 overexpressing RH4 cells is relevant when compared to the increase in CMV-transfected cells. Statistical significance was assessed by the Student's *t* test: $**p \leq 0.001$ $***p \leq 0.0001$.

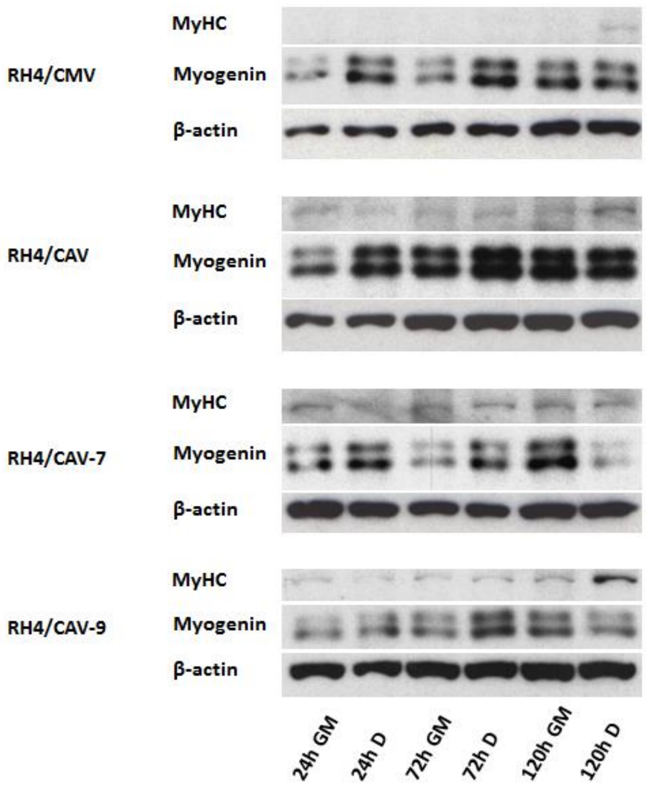

Supplementary Figure S7: Western blot showing myogenin and MyHC levels comparing normal growing conditions (GM) with differentiation conditions (D). Cells were growing for 24, 72 and 120 hours in both conditions.

Supplementary Table S1: Patient characteristics and CAV1 expression, as detected by immunohistochemistry, on a TMA of patients of rhabdomyosarcoma (n = 70). Legend: NA, not available; E, exitus; A, alive; L, local; M, metastasis.
